# Development of Fermented Sweet Potato Flour (*Ipomoea batatas* L.) Supplemented with Mackerel (*Scomber scombrus*) Meal-Based Biscuits

**DOI:** 10.1155/2022/8033978

**Published:** 2022-10-28

**Authors:** Hippolyte Tene Mouafo, Germaine Yadang, Gabrielle Ofakem Sibozo, Ruth Edwige Kemadjou Dibacto, Laurette Blandine Mezajoug Kenfack

**Affiliations:** ^1^Centre for Food, Food Security and Nutrition Research, Institute of Medical Research and Medicinal Plant Studies, PO Box 13033, Yaoundé, Cameroon; ^2^Department of Food Science and Nutrition, National School of Agro-Industrial Sciences, University of Ngaoundéré, PO Box 455, Ngaoundéré, Cameroon; ^3^Department of Food Engineering and Quality Control, University Institute of Technology, University of Ngaoundéré, PO Box 454 Ngaoundéré, Cameroon

## Abstract

The fermentation of sweet potato (*Ipomoea batatas* L.) with a strain of *Lactobacillus plantarum* leads to an increase in its exopolysaccharides (EPS) content which is useful for enhancing the functional properties of flour. The objective of this study was to develop healthy and nutritious meal-based biscuits from fermented sweet potato (FSP) flour supplemented with mackerel flour. Eleven formulations containing wheat flour, FSP flour, nonfermented sweet potato (NFSP) flour, and mackerel flour at different proportions defined following a mixture design were used to prepare biscuits. Physicochemical, microbiological, and sensory analyses of the different biscuits were performed. Biscuits prepared with FSP at 100% scored the lowest lipid (10.83 ± 0.97 g/100 g DM) and the highest sugar (67.43 ± 0.64 g/100 g DM) contents. The incorporation of mackerel flour in the formulation led to a significant (*p* < 0.05) increase in the protein and mineral contents of biscuits thus conferring an immune-boosting property to these latters. All the biscuits were of good microbiological quality independent of the formulation. The highest DPPH free radicals scavenging activity (IC_50_ of 1.90 and 3.41 *μ*g/mL for ethanolic and methanolic extracts, respectively) were observed with biscuits prepared with FSP flour at 100%. The sensorial characteristics of biscuits prepared with equal proportions of wheat and FSP flours were highly appreciated by the panelists with scores close to the ones prepared with wheat flour at 100%. The results of this study demonstrate the potential of FSP flour as a substitute for wheat flour in biscuits preparation. It also suggests biscuits prepared with FSP flour supplemented with mackerel flour as a functional and immune-boosting food.

## 1. Introduction

Nowadays, the demand for safe and nutritious diets satisfying energy needs and nutrition deficiency is constantly growing. To satisfy that demand, the development of new products has become the main concern of food industries [1]. Hence, new and innovative products are constantly introduced to markets, and the markets' part of these products is difficult to be measured [2]. Biscuits are amongst the main newly developed products highly prized by consumers. According to Hasker et al. [3], the most consumed human snacks in many parts of the world are biscuits. They are generally made using wheat flour as the main raw material. However, some limitations associated with the use of wheat flour, such as gluten intolerance, the cultural conditions of wheat, and its importation costs, have led to the development of research aiming at substituting wheat flour with other locally available food matrices. Several studies have demonstrated that flours from roots and tubers can be used to substitute wheat flour in biscuits preparation [4].

Sweet potato (*Ipomoea batatas* L.) stands as an example of these roots and tubers due to its easy cultural conditions (a low nutritional requirement for its growth), its nutritional value, and its richness in bioactive compounds like flavonoids, anthocyanins, *β*-carotenes, phenolic compounds, and vitamin C [5]. The carbohydrate, fiber, vitamins A and C, thiamine, riboflavin, niacin, and mineral (potassium, calcium, magnesium, sodium, phosphorus, and iron) contents of sweet potato can cover the human nutritional and energy needs [6]. The low cost of sweet potato and its nutritional values confer to this latter the ability to be used for reducing food insecurity and poverty [6]. As a good source of fibers, vitamins A and C, and bioactive compounds, the consumption of sweet potato might be associated with the prevention of cardiometabolic diseases [5, 6]. Sweet potato is largely produced in the world and particularly in Cameroon, and its use will reduce costs associated to the importation of wheat flour. In the literature, there are researches highlighting the use of sweet potato in biscuit preparation [7]. However, like flours from other roots and tubers that do not contain proteins which can form the network that will retain the gas produced during fermentation like gluten [8], the bakery products prepared with sweet potato flour do not swell and have a low loaf volume [9]. In a study conducted by Panyoo et al. [10], it was demonstrated that the strain produced a high amount of exopolysaccharides (EPS) during the fermentation of sweet potato by *Lactobacillus plantarum*. The swelling property of EPS reported in the literature improves the functional properties of flour [11] and might confer to fermented sweet potato (FSP) flour, a baking ability. Moreover, the antioxidant activity of EPS might also confer to biscuits prepared from FSP flour health benefits [12].

To improve the nutritional value of biscuits, they are generally supplemented. According to Ahmad and Ahmed [13], the supplementary ingredients should provide the necessary amount of nutrients taken in small quantities. Taking into consideration the COVID-19 context where immune-boosting foods are recommended, associated with the context of malnutrition in developing countries as well as in Cameroon, the choice of a supplementary ingredient was directed towards mackerel (*Scomber scombrus*) due to its richness in minerals such as zinc, unsaturated fatty acids, and proteins [14]. Hence, biscuits prepared from fermented sweet potato and supplemented could not only be nutritious and immune-boosting foods but also a functional food that might protect consumers against chronic diseases. The present study was designed and has as objective to develop healthy and nutritious meal-based biscuits from sweet potato flour supplemented with mackerel flour.

## 2. Materials and Methods

### 2.1. Raw Materials and Microbial Culture

The yellow variety of sweet potato (*Ipomoea batatas* L.) was used in this study. That variety was chosen because it is a *β*-carotenes fortified variety highly cultivated, prized, and consumed by the whole class of Cameroonians [15]. A total of 15 kg of mature samples of the yellow variety of sweet potato with approximately 250 g each was randomly collected in the city of Yaoundé, Cameroon, and channeled to the laboratory for analyses. Five samples of raw mackerels (*Scomber scombrus*) of approximately 500 g each were sampled in a fish shop, introduced in an icebox, and transported to the laboratory.

A strain of *Lactobacillus plantarum* for which previous studies demonstrated its ability to produce exopolysaccharides while using yellow sweet potato as substrate [10] was provided by the Laboratory of Microbiology of the University of Yaoundé I (Cameroon). From frozen stock, the strain was subcultured twice in milk broth (composition for 1 L of broth, skimmed milk: 40 g, glucose: 20 g, tween 80: 4 g) before being used for analyses.

### 2.2. Fermented Yellow Sweet Potato Flour Production

Samples of yellow sweet potato were superficially cleaned with tap water, disinfected with chlorine (0.2%, *v*/*v*), and rinsed with sterile distilled water. The tubers were peeled and cut manually with stainless steel in slices of 5 mm thickness. The slices were pooled, heated at 100°C for 5 min, and allowed to cool at room temperature (25 ± 2°C) in aseptic conditions. The precooked slices were fermented by the strain *L. plantarum* following the method described by Panyoo et al. [10]. Briefly, in aseptic conditions, 4 kg of the precooked potato slices was ground and inoculated with 200 mL of a culture of *L. plantarum* at 8 Log CFU/mL. The mixture was homogenized and incubated at 37°C for 33 h. After fermentation, the mixture was dried at 50°C for 72 h in a ventilated oven (Memmert, Germany), ground and sieved (*Ø* = 500 *μ*m). The flour of fermented sweet potato was packaged in a polyethylene bag and stored at 4°C for analyses. A sample of sweet potato treated in the same conditions but not inoculated with *L. plantarum* was used as a control to produce the nonfermented flour of sweet potato.

### 2.3. Production of Mackerel Flour

The fish species used in this study was *Scomber scombrus* commonly called mackerel. It was chosen based on its nutritional composition, its availability, and its affordability (low cost). The mackerel flour was produced following the method described by Murali et al. [16] with some modifications. Briefly, samples of fresh mackerels were descaled, eviscerated, and washed with tap water. Then, using a stainless steel knife, the fish was split open from the ventral side and minced into fillets of approximately 5 mm in thickness. Fillets were washed with tap water to remove adhering blood and rinsed with distilled water. Clean fillets were soaked in 1 L of a NaCl solution (250 g/L) and incubated at 10°C for 24 h. The fillets were laid on the racks of a ventilated oven and dried at 45°C for 48 h. The dried fillets were ground and sieved (*Ø* = 500 *μ*m) to obtain flour (821.62 g) which was packaged in a polyethylene bag and stored at 4°C for analyses.

### 2.4. Proximate Analysis of Biscuit Ingredients

The moisture and ash contents of flours were determined following the method AOAC [17]. Total sugars were determined according to Dubois et al. [18]. Lipids were assessed using the method of Bourely [19], while proteins were assessed using the method of Devani et al. [20].

### 2.5. Functional Properties of the Flours

The method of Philips et al. [21] was used to assess the water holding capacity of flour samples. The water solubility index and the oil holding capacity of flour samples were determined following the methods described by Anderson et al. [22] and Sosulski [23], respectively.

### 2.6. Baking and Biscuit Processing

The method described by Eyenga et al. [24] with some modifications was used to produce biscuits. The ingredients used for biscuits preparation were fermented sweet potato flour, mackerel flour, wheat flour, sugar, margarine, eggs, and baking powder. In the protocol, sugar (SOSUCAM, Mbandjock, Cameroon) and margarine (Jadida, Yaoundé, Cameroon) were introduced into a clean bowl. The mixture was creamed thoroughly until obtaining snow and smooth homogenous mixture. Eggs (Yaoundé, Cameroon) were added to the mixture followed by stirring to obtain a creamy paste. Then, the mixture of flours (wheat, fermented sweet potato, and fish following the design mentioned in Table 1), baking powder, and salt were sieved (500 *μ*m) and added to the creamy paste. The mixture was thoroughly homogenized with a spatula. The dough obtained was cut and shaped using a biscuit mould (0.5 cm thick discs of 6 cm diameter). The shaped doughs were deposited on a clean stainless steel tray previously greased with margarine. The trays containing the shaped dough were introduced into a Memmert electric oven (Memmert IN110, Germany) and baked at 180°C for 15–20 min. The biscuits were removed from the oven and cooled at room temperature (25 ± 2°C) before being packaged in polyethylene bags. To determine the quantities of wheat flour, fermented sweet potato flour, and mackerel flour leading to a biscuit with the best microbiological, physicochemical, and sensorial qualities, eleven formulations with variable quantities of these ingredients were generated using Minitab version 18.1 (Minitab Inc., USA). The different formulations are presented in Table 1.

For the proximal composition of the different samples of biscuits, moisture and ash were determined following the method AOAC [17]. Total sugars were determined according to Dubois et al. [18]. Lipids were assessed using the method of Bourely [19], while proteins were assessed using the method of Devani et al. [20]. The energetic value of biscuits was evaluated using the method proposed by AFNOR [25].

### 2.7. Microbiological Validation of Processed Biscuits

Samples were prepared following the normalized method ISO 6887-2 [26]. For each biscuit, 100 g was crushed in aseptic conditions, and 25 g was taken and transferred into a sterile flask containing 225 mL of sterile saline (NaCl 0.85%). The mixture was homogenized and left at room temperature (25 ± 2°C) for 30 min. Then, serial dilutions were made (10^−1^ to 10^−6^). The total aerobic mesophilic flora was enumerated on Plate Count Agar (PCA, LiofilChem, Italy) following the pour plate method ISO 4833-1 [27]. Yeast and mould enumeration was performed on Sabouraud agar supplemented with chloramphenicol (LiofilChem, Italy), using the method ISO 21527-1 [28]. The method ISO 6888-2 [29] was used to enumerate *Staphylococcus* spp. while total coliforms and *E. coli* were enumerated following the method ISO4832 [30]. Following incubation, colonies forming units appearing in the Petri dishes were counted. The microbial counts obtained from triplicate experiments were summarized as mean loads, and the final results were expressed as colonies forming units per gram of biscuits.

### 2.8. Sensory Evaluation of Biscuits

The sensory characteristics of the 11 biscuits produced were evaluated by 20 trained panelists (12 women and 8 men) following the method described by Eyenga et al. [24] with some modifications was used. The selection criteria of these panelists were their regular frequency of consumption of biscuits, good health, nonsmoker, availability, and nonallergy to any of the ingredients used in the preparation of biscuits. They were informed of the objectives, and only volunteers were recruited. Before the beginning of the test, all the panelists signed an informed consent sheet. In the experimental procedure, biscuits were assessed using a 9-point scale ranging from “0 = extremely dislike” to “9 = extremely like.” The descriptors assessed by the panelists were color, crispness, odor, taste, and overall acceptability. Samples were codded and randomly presented to the panelists. Following the description of the intensity of the different descriptors of one biscuit, panelists were asked to rinse their mouth with water before continue to the next one.

### 2.9. DPPH Radical Scavenging Activity of Processed Biscuits

The antioxidant activity of biscuits produced in this study was assessed through their ability to scavenge free radicals. For this, the DPPH (2.2-diphenyl-1-picrylhydrazyl) free radical scavenging method described by Uddin et al. [31] was used. Aqueous, ethanolic, and methanolic extracts of biscuits were prepared at 6 concentrations (10, 6.7, 3.4, 1.6, 0.83, and 0.05 *μ*g/mL), and 1 mL of each solution was mixed with 4 mL of DPPH solution (0.2 mM in methanol). The mixture was homogenized and stored in darkness at room temperature (25 ± 1°C) for 30 min. Then, the optical density was read at 536 nm against the blank (solvent). The DPPH radical scavenging activity was calculated using the formula:

(1) $$\begin{array}{c} {\text{DPPH}}_{\text{scavenging activity }\left(\%\right)}=\frac{\left({\text{OD}}_{\text{control}}-{\text{OD}}_{\text{sample}}\right)}{{\text{OD}}_{\text{control}}}\times 100, \end{array}$$

where OD_control_ refers to the optical density of the solution free of extract, and OD_sample_ refers to the optical density of the solution containing extract at a given concentration.

The concentration providing 50% of DPPH inhibition (IC_50_) was calculated from a graph representing the DPPH inhibition percentage as a function of the biscuit concentrations. It was used to compare the antioxidant activity of the different biscuit samples. Ascorbic acid (vitamin C) was used as standard.

### 2.10. Statistical Analyses

Experiments were performed in triplicated, and the results are presented as means ± standard deviation. Analysis of variance and Duncan multiple range test were performed to compare means at a statistical significance of 5%. Graphs were plotted using Sigma Plot 12.5 version 12.5.0.38 (Systat Software, Inc., Chicago IL, USA).

## 3. Results and Discussion

### 3.1. Production of Flours

Flour production yields of 75.88, 66.66, and 64.61% were obtained from fermented sweet potato, nonfermented sweet potato, and mackerel, respectively. A granulometric distribution of fermented sweet potato flour revealed that its particles' size was between 200 and 500 *μ*m. These particles' sizes were higher than that of wheat flour which varies between 50 and 200 *μ*m. This result suggests that the use of fermented sweet potato flour in biscuit production might lead to a more granular biscuit. A similar observation was made by Eyenga et al. [24] as the authors noticed an increase in the granulometry of biscuits following the addition of safou (*Dacryodes edulis*) powder.

### 3.2. Proximate Analysis of Biscuit Ingredients

Globally, the flour from FSP scored the highest water content (12.75 ± 0.27%) while the least one was recorded with wheat flour (6.70 ± 0.22%). The significant difference (*p* < 0.05) in water content observed between the flours from fermented (12.75 ± 0.27%) and nonfermented (8.08 ± 0.40%) sweet potato could be ascribed to the presence of exopolysaccharides (EPS) produced by the strain *L. plantarum* during the fermentation [10]. Indeed, due to their structure, EPS can fix and trap water molecules with as consequence an increase in the water content [32]. The ash content of flours from fermented (2.10 ± 0.20 g/100 g DM) and nonfermented (2.25 ± 0.30 g/100 g DM) sweet potato was not significantly different (*p* > 0.05), thus, demonstrating that the fermentation of sweet potato by *L. plantarum* did not influence its mineral content. The ash content of wheat flour (0.87 ± 0.04 g/100 g DM) was significantly (*p* < 0.05) lower than that of sweet potato flour. This difference arises from their physiologic growth conditions and their mineral composition.

### 3.3. Functional Properties of the Flours

Some functional characteristics of sweet potato flours were assessed, and the results are presented in Table 2. The flour from FSP showed the highest water holding capacity (741.39 ± 19.29%) compared to NFSP flour (487.94 ± 3.83%) and wheat flour (77.06 ± 4.05%). This could be attributed to the EPS present in FSP flour. Saravanan and Shetty [32] also reported that EPS from *Leuconostoc lactis* KC117496 deserved high water holding capacity. Moreover, another explanation for the increase in water holding capacity might be the fact that during the fermentation of sweet potato, and the peptide bonds of proteins are broken through the proteolytic activity of *L. plantarum* leading to an increase in the number of polar or hydrophilic groups of the flour proteins as highlighted by Adams et al. [33]. The values of water holding capacity of sweet potato flours obtained in this study were higher than 210% reported by Ndangui [34] with sweet potato flours. The difference could be explained by the production process of flour. In this study, the preheating unit operation applied to the sweet potato before drying could have improved the water holding capacity of sweet potato starch. An increase in the water holding capacity as a result of the pregelatinization treatment applied to Gayam (*Inocarfus fagifer*) flour was noticed by Wijanarka et al. [35]. It was reported that bakers like more flour with higher water holding capacity as it enables the addition of more water to the dough [36]. Hence, the high water holding capacity of FSP flour obtained in this study suggests the suitability of this matrix in the bakery as a substitute for wheat flour.

The water solubility index of sweet potato flours was also assessed. While wheat flour showed the least value of water solubility index (25.43 ± 1.84%), no significant (*p* > 0.05) difference was noticed between the water solubility index of flours from fermented (54.53 ± 0.15%) and nonfermented (56.36 ± 1.61%) sweet potato. The high values of the water solubility index obtained with sweet potato flours could be explained by the difference in starch structure. Indeed, during the preheating treatment applied to sweet potato, the structure of starch granules changed leading to an increase in their water solubility [37].

Another important functional property of flours assessed in this study was their oil holding capacity since oils can improve the mouth feel of products as well as their retention of flavors [38]. Regarding the oil holding capacity, the fermentation has led to a significant (*p* < 0.05) decrease in the oil holding capacity of sweet potato flour. This could be ascribed to the reduction of nonpolar compounds (nonpolar starch chains, nonpolar amino acids, etc.) during the fermentation of sweet potato. Adams et al. [33] also found that the fermentation of sweet potato reduced its oil holding capacity.

### 3.4. Proximate Composition of the Different Biscuits

Table 3 depicts the physicochemical characteristics of the 11 different biscuits produced in this study. The lowest values of water content were recorded with biscuits BS2, BS5, and BS9. It corresponds to biscuits with high proportions of wheat flour. This observation could be attributed to the weakly water content of wheat flour as previously observed (Table 2). As the proportion of FSP flour in the mixture increases, the water content of biscuits increases significantly (*p* < 0.05). The incorporation of mackerel flour in the biscuit formulation has not shown a significant (*p* < 0.05) effect on their water contents. The highest water content (13.98 ± 1.33%) was recorded with biscuit BS6 prepared only with FSP flour. Its counterpart biscuit BS10 prepared only with NFSP flour scored a water content of 11.05 ± 0.26%. This could be attributed to the high water holding capacity of the flour from FSP as shown previously. According to FAO [39], the water content of biscuits intended to be stored for a long period should not exceed 5%. The highest water content of biscuits prepared with FSP flour suggests a reduced shelf life for these products and requires that these biscuits should be handled with respect of good hygiene practices.

Low lipid contents were recorded with biscuits BS4 (10.83 g/100 g DM), BS6 (11.46 g/100 g DM), and BS7 (11.98 g/100 g DM). These biscuits were characterized by their high proportions of flours from FSP. However, as the proportion of FSP flour decreases, a significant (*p* < 0.05) increase in lipid contents of biscuits was noticed. This can be justified by the low oil holding capacity of flour from FSP. Hence, during the baking process, lipids are not held in the dough leading to a reduction of lipids' contents in the biscuits. A similar observation was noticed by Nguyen and Nguyen [40]. The highest lipid content was noticed with biscuit BS11 (25.96 g/100 g DM) made with an equal proportion of wheat flour and flour from NFSP. This could be ascribed to the highest values of oil holding capacity of these flours. The presence of mackerel flour in that biscuit suggests an improvement in the quality of its fatty acid profile as mackerel contained unsaturated fatty acids such as omega 3 and 6. Although high lipid content is important for biscuits as they contributed to improving mouth feeling and the retention of flavors, they might also contribute to its spoilage through the lipid oxidation process and, thus, reduce their shelf life. The lipid contents of biscuits obtained in this study are in accordance with 25.38 ± 0.02 g/100 g DM reported by Eyenga et al. [24] with biscuits made with safou.

The protein contents of the different samples vary significantly (*p* < 0.05) from one biscuit to another. Globally, the protein content of biscuits was found to increase with the proportion of mackerel flour and decrease when the proportion of FSP flour decreases. This observation can be attributed to the highest protein content of mackerel flour as reported in the literature [14]. The highest protein content (19.35 ± 0.06 g/100 g DM) was recorded with biscuit BS5 made with wheat (95%) and mackerel (5%) flours while the lowest one (6.49 g/100 g DM) was noticed with biscuit BS4 made with flour from FSP at 100%. The utilization of proteins by the strain *L. plantarum* through metabolic activity leading to the production of EPS during the fermentation of sweet potato could explain this result. However, it is important to notice that biscuits BS7 prepared with 74.38% of FSP flour, 1.25% of mackerel flour, and only 24.37% of wheat flour scored a high protein content of 10.91 g/100 g DM. This observation demonstrates the suitability of FSP flour as a substitute for wheat flour in the production of biscuits that could be consumed by persons independent of their age to fight against malnutrition. The protein contents of biscuits prepared with flour from FSP were generally higher than 10.13 ± 0.80 g/100 g DM reported by Eyenga et al. [24] with biscuits made with safou as the substitute for wheat flour. This difference could be ascribed to the low level of protein in safou compared to mackerel.

Sugars were present in all biscuits at levels that range significantly (*p* < 0.05) from 47.17 g/100 g DM (BS5) to 66.43 g/100 g DM (BS4). This high proportion of total carbohydrates in biscuits could result from the chemical composition of flours. The results of the present study demonstrate the high energetic potential of the different biscuits and suggest their suitability for children. Indeed, the quality of food formulated with as target adults or children for whom the energy requirements are high is generally assessed through their energy content [41]. In this study, the energetic values of the different biscuits were assessed, and the results presented in Table 3 show that biscuit BS8 scored the least energetic value of 302.56 kcal/100 g DM. The highest value (490.74 kcal/100 g DM) was recorded with biscuit BS9. A deep observation of Table 3 revealed that the energetic value decreases as the proportion of flour from FSP increases. This can be ascribed to the low oil holding capacity of the FSP flour leading to a low lipid content in the biscuit and, thus, a low energetic value.

As observed in Table 3, the ash content of the different biscuits was proportional to their mackerel flour content. The highest values of 5.15 and 5.84 g/100 g DM were noticed, respectively, with biscuits BS1 and BS5 where the proportion of mackerel flour was maxima (5%). The results of this study demonstrate the suitability of mackerel flour as a potential source of minerals and suggest the use of biscuits containing mackerel flour as an immune-boosting product that can be used in this context of the COVID-19 pandemic. Indeed, mackerel is a good source of zinc and iron [14]. Zinc is essential for immune cells to activate macrophages and neutrophils. It has a beneficial effect on intestinal immune function, restores thymulin activity, and reduces the number of activated helper T cells (which might contribute to autoimmunity) [42]. Apart from these immune-boosting properties, zinc also inhibits the RNA polymerase of viruses such as SARS-CoV-2 [42]. Iron was reported to deserve immune-boosting property through its ability to activate the proliferation of lymphocytes [42].

Besides mackerel flour, FSP flour appears as an important source of minerals compared to wheat flour. Indeed, the ash contents of biscuits prepared with FSP flour were higher than that of wheat flour. This observation can arise from the fact that FSP flour contained more ash (2.10 g/100 g DM) compared to wheat flour (0.87 g/100 g DM).

### 3.5. Microbiological Quality of Biscuits

The results of the microbiological analysis of the different samples of biscuits are presented in Table 4. The different biscuits scored a total aerobic mesophilic count ranging significantly (*p* < 0.05) from 1.33 ± 0.03 to 3.00 ± 0.01 Log CFU/g. The most contaminated sample was biscuit BS1 (3 Log CFU/g). Globally, there was no association between the proportions of flour from FSP and the TAMF loads. Compared to the norms for which the TAMF count of biscuits should not exceed 3 Log CFU/g [43], all biscuits were suitable for human consumption. Pathogens such as *E. coli* and total coliforms were absent in all samples (Table 4). Although *Staphylococcus* spp. were detected in biscuits BS1 (0.77 ± 0.10 Log CFU/g), BS2 (0.53 ± 0.08 Log CFU/g), BS3 (0.81 ± 0.05 Log CFU/g), and BS7 (0.87 ± 0.04 Log CFU/g), their loads were all lower than the threshold value recommended by the European Commission regulation (1 Log CFU/g) [43]. Yeasts and moulds were found in all samples of biscuits with loads varying from 0.69 ± 0.12 Log CFU/g (BS9) to 1.27 ± 0.03 Log CFU/g (BS1). Their presence in the biscuits could be ascribed to their spore-forming ability. That property of this group of microorganisms is responsible for their resistance to heat treatment applied during baking. Another explanation could be postcontamination of biscuits following their air exposition for cooling purpose. Considering the norms [43], the loads in yeasts and moulds of the different samples were below 2 Log CFU/g, thus justifying their suitability for human consumption.

### 3.6. Sensory Quality of the Different Biscuits

**Figure 1:**
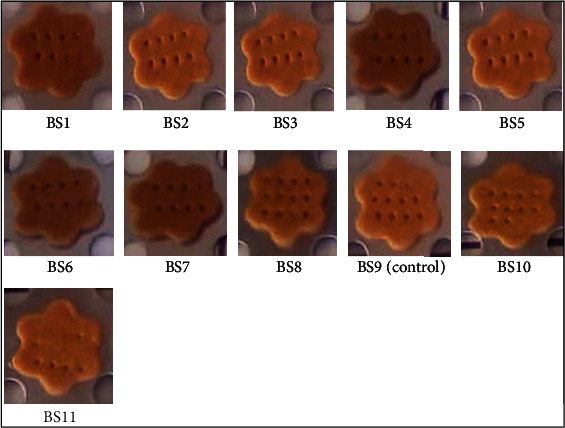
Biscuit samples prepared with wheat, fermented sweet potato, nonfermented sweet potato, and mackerel flours at different proportions.

For newly developed food products like biscuits prepared with FSP and mackerel flours, the descriptive sensory analysis appears as a powerful tool to predict its acceptance by consumers. The biscuits prepared with FSP flour at different proportions are depicted in Figure 1. These biscuits were submitted to sensory evaluation by the panelists.

For the development of new products intended for human consumption, color is generally used as the criteria that mostly influences the purchasing decision. In this study, the color of biscuits was assessed by the panelists, and biscuits BS5 and BS9 show the highest scores of 7.90 ± 1.16 and 7.55 ± 0.99, respectively (Table 5). These biscuits were free of flour from FSP. This can be justified by the fact that panelists more appreciated biscuits that the color tends to yellow as they look like the major biscuits commonly found in markets. The biscuit prepared only with FSP flour presented the lowest score (BS4). The dark brown color of biscuits prepared with the FSP flour (Figure 1) that was not appreciated by some panelists might result from the presence in FSP flour of a huge amount of reducing sugars that were caramelized during the baking process [44].

Besides color, one of the desirable textural criteria of biscuits generally appreciated by consumers is their crispness [24]. In this study, the crispness of biscuits decreases as the proportion of FSP flour in the formulation increases (Table 5). This can be justified by the highest water contents of these biscuits and their rapid tendency to absorb humidity and become hard due to their high contents in reducing sugars. Indeed, these biscuits were hard, and some forces were required for their breakage compared to others. Associated to their hardness, the sound emitted during that breakage process was weak. Comparing BS5 (95% wheat flour and 5% mackerel flour) and BS9 (100% of wheat flour), it seems like the incorporation of mackerel flour slightly reduced the crispness of biscuits (Table 5).

Odor of the biscuits was also evaluated by the panelists. Globally, although nonsignificant (*p* > 0.05), the odor scores increase as the proportion of FSP flour decreases. The highest scores were recorded with biscuits prepared with wheat flour and free of sweet potato flour (BS5 and BS9). This can be explained by the low oil retention ability of the FSP flour as it is well known that lipids contribute to improving the retention of flavors. Hence, wheat flour with its high oil retention capacity will contain more flavors developed during the baking process. Another explanation for the fact that panelists have mostly appreciated the biscuit BS5 prepared with wheat and mackerel flours could be the specific aromatic compounds brought by the mackerel flour.

With regards to taste, the most appreciated biscuit was BS9 (7.90) prepared with wheat flour at 100%. Surprisingly, biscuit BS4 prepared with 100% FSP flour also showed a high taste score of 7.80. This appreciation of their taste by the panelists could be attributed to the high sugar content of these biscuits as starch was hydrolyzed into reducing sugars during the fermentation [12]. Moreover, the acidic nature of these biscuits and their specific flavor that result from the fermentation with *L. plantarum* could also justify the scores obtained. Taking into consideration the mackerel flour, although nonsignificant (*p* > 0.05), the taste globally decreases as its proportion in the biscuits decreases. This result suggests that the incorporation of the mackerel flour in the dough formulation might bring an improvement in the final taste of biscuits.

Regarding the overall acceptability, the least appreciated biscuit was BS7 with a score of 5.36. Biscuits prepared with FSP flour at 100% (BS4) showed a weak overall acceptability score. The highest overall acceptability score (8.10) was recorded with biscuit BS9 prepared with 100% of wheat. Srivastava et al. [43] also reported following a sensorial evaluation by the panelists, a higher preference score for wheat-based biscuits is compared to the sweet potato-based ones. Biscuit BS8 prepared with equal proportions of wheat and FSP flours was also highly appreciated (7.95) with a score close to the one obtained with wheat flour at 100% (BS9). This result suggests the application of response surface methodology through optimization design to determine the proportions of the mixture that will lead to maximum overall acceptability.

**Figure 2:**
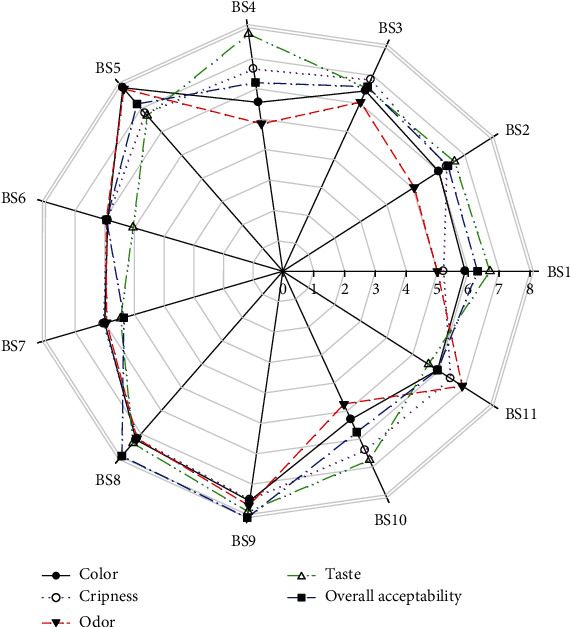
Radar plot showing the sensorial parameters of the different biscuits.

Figure 2 summarizes the scores of the different sensory descriptors of biscuits that were evaluated by the panelists. It can be observed that the most appreciated biscuits were BS5, BS8, and BS9.

### 3.7. Antioxidant Activity of Biscuits

**Figure 3:**
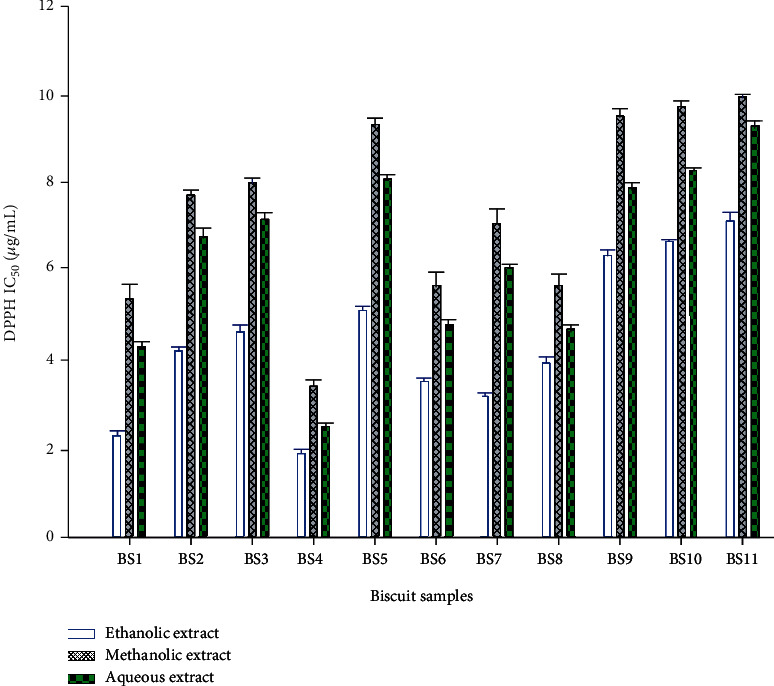
DPPH IC_50_ of the ethanolic and methanolic extracts of the different biscuits.

In this study, the properties of EPS produced by *L. plantarum* in sweet potato paste during fermentation were exploited as a swollen baking agent to confer the same characteristics as wheat flour to sweet potato flour. Besides this baking ability, the antioxidant activity of the aqueous, ethanolic, and methanolic extracts of the different biscuits containing these EPS was assessed through their ability to scavenge DPPH free radicals. This test was chosen because free radicals are the main cause of the imbalance of antioxidants leading to oxidative stress in the human body. DPPH free radical was reported to act as an extensively proven tool for evaluating the ability of antioxidants to scavenge free radicals [45]. All the extracts deserved DPPH free radicals scavenging activities independent of the solvents used. Globally, the activity was proportional to the extract concentration and varied significantly (*p* < 0.05) with the biscuits and the solvents. The IC_50_ (concentration of the extract required to scavenge 50% of DPPH free radicals) of the different extracts were determined and the results are presented in Figure 3. The IC_50_ values of the different extracts ranged from 1.90 ± 0.02 to 9.95 ± 0.07 *μ*g/mL and were all higher than that of the standard ascorbic acid (0.021 ± 0.001 *μ*g/mL).

With regards to the biscuits, the antioxidant activity generally increases when the proportion of FSP flour in the formulation increases. The extracts from biscuit BS4 (100% FSP flour) were more active as they scored the lowest IC_50_ values. This can be attributed to their high contents in EPS. Several studies have demonstrated the antioxidant activity of EPS [12]. Indeed, the hydroxyl groups of EPS can donate active hydrogen to DPPH free radicals leading to their scavenging [45]. Besides EPS, sweet potato also contained bioactive compounds with demonstrated antioxidant activities such as flavonoids (quercetin, kaempferol, catechins, and anthocyanidins), polyphenols, and *β*-carotene [46]. This result suggests that biscuits prepared with FSP flour can be used as a functional food for the management of chronic diseases such as cardiovascular diseases, obesity, and hypertension.

Considering the extraction solvents, ethanolic extracts were more active than aqueous and methanolic extracts independent of the biscuits as significant differences (*p* < 0.05) in IC_50_ values were noticed (Figure 3). This can be ascribed to the fact that ethanol extracted more polysaccharides compared to methanol [47]. These polysaccharides including EPS might globally be responsible for the antioxidant activity of biscuits. Besides, the highest IC_50_ values recorded with aqueous extracts compared to the methanolic ones also justify the fact that polar compounds of biscuits such as polysaccharides could be mostly involved in the antioxidant activity.

## 4. Conclusion

This study demonstrated the ability of FSP flour to be used as a swollen baking agent in biscuit preparation and suggests its potential as a substitute for wheat flour. Besides their nutritional values, biscuits prepared with FSP flour deserve functional properties and can be suitable for the management of chronic diseases. The incorporation of mackerel flour in the formulation has led to a significant improvement in the protein and mineral contents of the biscuits thus conferring an immune-boosting property to these latters. Biscuits formulated with equal proportions of wheat and FSP flours were highly appreciated by the panelists. However, given that some sensorial characteristics of biscuits prepared with the FSP flour at 100% were not highly appreciated by the panelists, further studies should be performed to optimize the formulation and characterize the EPS.

## Figures and Tables

**Table 1:** Different formulations of flours used for biscuits preparation.

Formulations	Wheat flour (g/100 g)	FSPF (g/100 g)	Mackerel flour (g/100 g)	NFSPF (g/100 g)
Biscuit BS1	0	95	5	0
Biscuit BS2	71.87	24.38	3.75	0
Biscuit BS3	74.37	24.38	1.25	0
Biscuit BS4	0	100	0	0
Biscuit BS5	95	0	5	0
Biscuit BS6	24.37	71.88	3.75	0
Biscuit BS7	24.37	74.38	1.25	0
Biscuit BS8	48.75	48.75	2.5	0
Biscuit BS9 (control)	100	0	0	0
Biscuit BS10	0	0	0	100
Biscuit BS11	48.75	0	2.5	48.75

FSPF: fermented sweet potato flour; NFSPF: nonfermented sweet potato flour.

**Table 2:** Water holding capacity, water solubility index, and oil holding capacity of flours from wheat, fermented, and nonfermented sweet potato.

Samples	WHC (%)	WSI (%)	OHC (%)
Wheat flour	77.06 ± 4.05^a^	25.43 ± 1.84^a^	69.86 ± 1.84^a^
Fermented sweet potato flour	741.39 ± 19.29^c^	54.53 ± 0.15^b^	63.50 ± 7.27^a^
Nonfermented sweet potato flour	487.94 ± 3.83^b^	56.36 ± 1.61^b^	81.93 ± 3.00^b^

WHC: water holding capacity; WSI: water solubility index; OHC: oil holding capacity. Values bearing different superscript letters on the same raw are significantly different (*p* < 0.05) according to Duncan's multiple range test.

**Table 3:** Different formulations of biscuits: proximate composition (g/100 g DM) and energetic values (kcal/100 g DM).

Biscuit samples	Water content	Lipids	Proteins	Total sugars	Ash	Energetic value
BS1	12.20 ± 0.18^de^	16.30 ± 0.19^d^	12.59 ± 0.63^c^	53.77 ± 0.04^b^	5.14 ± 0.50^i^	412.14 ± 0.98^d^
BS2	5.71 ± 0.64^a^	21.72 ± 0.63^g^	12.60 ± 0.07^c^	56.11 ± 0.50^c^	3.77 ± 0.20^g^	470.32 ± 4.01^g^
BS3	10.63 ± 0.08^c^	19.24 ± 0.98^f^	12.00 ± 0.90^c^	55.47 ± 0.09^c^	2.66 ± 0.01^d^	443.04 ± 4.86^f^
BS4	13.98 ± 1.33^e^	10.83 ± 0.97^a^	6.49 ± 1.64^a^	66.43 ± 0.64^f^	2.27 ± 0.02^c^	389.15 ± 4.77^b^
BS5	5.25 ± 0.12^a^	22.39 ± 0.13^h^	19.35 ± 0.06^d^	47.17 ± 0.20^a^	5.84 ± 0.04^j^	467.59 ± 0.69^g^
BS6	12.18 ± 0.60^de^	11.46 ± 0.39^a^	11.20 ± 0.80^c^	61.03 ± 1.45^d^	4.26 ± 0.01^h^	392.06 ± 2.01^b^
BS7	10.42 ± 0.46^c^	11.98 ± 0.12^b^	10.91 ± 1.18^c^	63.50 ± 1.05^e^	3.19 ± 0.01^e^	405.46 ± 0.55^c^
BS8	9.34 ± 0.08^b^	14.48 ± 0.98^c^	12.24 ± 1.74^d^	59.78 ± 0.74^d^	4.16 ± 0.10^h^	302.56 ± 4.89^a^
BS9 (control)	4.91 ± 0.54^a^	23.34 ± 0.31^i^	8.96 ± 1.07^b^	61.21 ± 1.10^d^	1.58 ± 0.33^a^	490.74 ± 2.93^h^
BS10	11.05 ± 0.26^d^	17.96 ± 0.30^e^	6.83 ± 0.41^a^	61.96 ± 0.87^d^	2.02 ± 0.04^b^	436.80 ± 1.34^e^
BS11	10.45 ± 0.15^c^	25.96 ± 0.50^j^	12.80 ± 1.83^c^	47.40 ± 1.15^a^	3.52 ± 0.17^f^	436.04 ± 1.85^e^

Values bearing different superscript letters on the same raw are significantly different (*p* < 0.05) according to Duncan's multiple range test.

**Table 4:** Microbial loads (Log UFC/g) of the biscuits prepared with different proportions of flour from fermented sweet potato.

Biscuit samples	TAMF	Yeasts and moulds	*Staphylococcus* spp.	Total coliforms	*E. coli*
BS1	3.00 ± 0.01^g^	1.27 ± 0.03^c^	0.77 ± 0.10^c^	0.00 ± 0.00^a^	0.00 ± 0.00^a^
BS2	2.17 ± 0.01^e^	1.07 ± 0.10^ab^	0.53 ± 0.08^b^	0.00 ± 0.00^a^	0.00 ± 0.00^a^
BS3	1.80 ± 0.03^d^	0.89 ± 0.07^a^	0.81 ± 0.05^cd^	0.00 ± 0.00^a^	0.00 ± 0.00^a^
BS4	1.44 ± 0.02^b^	1.06 ± 0.04^ab^	0.00 ± 0.00^a^	0.00 ± 0.00^a^	0.00 ± 0.00^a^
BS5	1.60 ± 0.01^c^	0.77 ± 0.10^a^	0.00 ± 0.00^a^	0.00 ± 0.00^a^	0.00 ± 0.00^a^
BS6	1.33 ± 0.03^a^	1.33 ± 0.11^c^	0.00 ± 0.00^a^	0.00 ± 0.00^a^	0.00 ± 0.00^a^
BS7	2.84 ± 0.21^fg^	1.10 ± 0.02^b^	0.87 ± 0.04^d^	0.00 ± 0.00^a^	0.00 ± 0.00^a^
BS8	2.47 ± 0.34^f^	1.05 ± 0.03^c^	0.00 ± 0.00^a^	0.00 ± 0.00^a^	0.00 ± 0.00^a^
BS9 (control)	1.68 ± 0.02^d^	0.69 ± 0.12^a^	0.00 ± 0.00^a^	0.00 ± 0.00^a^	0.00 ± 0.00^a^
BS10	1.46 ± 0.02^b^	1.11 ± 0.04^b^	0.00 ± 0.00^a^	0.00 ± 0.00^a^	0.00 ± 0.00^a^
BS11	1.37 ± 0.01^a^	1.20 ± 0.13^bc^	0.00 ± 0.00^a^	0.00 ± 0.00^a^	0.00 ± 0.00^a^
Norms (log UFC/g)	3	2	1	1	0

TAMF: total aerobic mesophilic flora. Values bearing different superscript letters on the same raw are significantly different (*p* < 0.05) according to Duncan's multiple range test.

**Table 5:** Sensory characteristics of the biscuits prepared with different proportions of flour from fermented sweet potato.

Biscuit samples	Color	Crispness	Odor	Taste	Overall acceptability
BS1	5.90 ± 1.16^ab^	5.20 ± 1.07^a^	5.00 ± 1.16^ab^	6.70 ± 1.17^b^	6.30 ± 1.26^ab^
BS2	6.00 ± 0.97^ab^	6.30 ± 1.30^ab^	5.05 ± 1.53^ab^	6.60 ± 1.18^b^	6.35 ± 1.30^ab^
BS3	6.45 ± 1.35^ab^	6.85 ± 1.18^ab^	6.05 ± 1.23^ab^	6.55 ± 1.09^b^	6.60 ± 0.75^ab^
BS4	5.55 ± 1.79^a^	6.65 ± 1.87^ab^	4.85 ± 0.55^a^	7.80 ± 1.07^b^	6.20 ± 1.53^a^
BS5	7.90 ± 1.16^b^	6.80 ± 1.15^ab^	7.85 ± 1.10^b^	6.70 ± 1.86^a^	7.20 ± 1.39^b^
BS6	5.90 ± 1.33^ab^	5.93 ± 1.62^a^	5.95 ± 1.09^ab^	5.05 ± 1.03^a^	5.94 ± 1.46^a^
BS7	6.00 ± 1.49^ab^	6.05 ± 1.50^ab^	5.94 ± 1.64^ab^	5.47 ± 1.74^a^	5.36 ± 1.57^a^
BS8	6.50 ± 1.14^ab^	7.20 ± 1.00^b^	7.20 ± 1.36^b^	7.40 ± 1.09^b^	7.95 ± 1.27^b^
BS9 (control)	7.55 ± 0.99^b^	7.50 ± 0.44^b^	7.70 ± 1.17^b^	7.90 ± 1.07^f^	8.10 ± 1.25^b^
BS10	5.90 ± 1.11^a^	6.40 ± 1.42^b^	4.75 ± 1.04^a^	6.75 ± 1.12^a^	5.75 ± 1.57^a^
BS11	5.95 ± 1.46^a^	6.45 ± 1.50^b^	6.90 ± 1.29^b^	5.60 ± 1.50^a^	5.95 ± 1.19^a^

Values bearing different superscript letters on the same raw are significantly different (*p* < 0.05) according to Duncan's multiple range test.

## Data Availability

The data used in this study are available from the corresponding author upon request.

## Abbreviations

CFU: Colony forming unit
DM: Dry matter
DPPH: 2.2-Diphenyl-1-picrylhydrazyl
EPS: Exopolysaccharides
EMB: Eosin methylene bleu agar
FSP: Fermented sweet potato
IC_50_: Concentration leading to 50% of DPPH inhibition
MSA: Mannitol salt agar
NaCl: Sodium chloride
NFSP: Nonfermented sweet potato
OD: Optical density
OHC: Oil holding capacity
PCA: Plate count agar
TAMF: Total aerobic mesophilic Flora
WHC: Water holding capacity
WSI: Water solubility index.
